# Comparison of pay-for-performance (P4P) programs in primary care of selected countries: a comparative study

**DOI:** 10.1186/s12913-023-09841-6

**Published:** 2023-08-14

**Authors:** Sara Jamili, Mehdi Yousefi, Hossein Ebrahimi pour, Elahe Houshmand, Ali Taghipour, Seyed Saeed Tabatabaee, Amin Adel

**Affiliations:** 1 Student Research Committee, Department of Health Economics and Management Sciences, Mashhad University of Medical Sciences, Mashhad, Iran; 2 Department of Health Economics and Management, School of Health, Mashhad University of Medical Sciences, Mashhad, Iran; 3 Department of Epidemiology and Biostatistics, School of Health, Mashhad University of Medical Sciences, Mashhad, Iran; 4 Health Management and Economics Research Center, Iran University of Medical Sciences, Tehran, Iran

**Keywords:** Payment methods, Pay-for-performance, Primary care

## Abstract

**Background:**

Pay for performance (P4P) schemes provide financial incentives or facilities to health workers based on the achievement of predetermined performance goals. Various P4P programs have been implemented around the world. There is a question of which model is suitable for p4p implementation to achieve better results. The purpose of this study is to compare pay for performance models in different countries.

**Methods:**

This is a descriptive-comparative study comparing the P4P model in selected countries in 2022. Data for each country are collected from reliable databases and are tabulated to compare their payment models. the standard framework of the P4P model is used for data analysis.

**Results:**

we used the standard P4P model framework to compare pay for performance programs in the primary care sector of selected countries because this framework can demonstrate all the necessary features of payment programs, including performance domains and measures, basis for reward or penalty, nature of the reward or penalty, and data reporting. The results of this study show that although the principles of P4P are almost similar in the selected countries, the biggest difference is in the definition of performance domains and measures.

**Conclusions:**

Designing an effective P4P program is very complex, and its success depends on a variety of factors, from the socioeconomic and cultural context and the healthcare goals of governments to the personal characteristics of the healthcare provider. considering these factors and the general framework of the features of P4P programs are critical to the success of the p4p design and implementation.

## Introduction

Pay for performance (P4P) schemes – also called performance-based financing or results-based financing schemes – provide financial incentives to health workers or facilities based on the achievement of pre-specified performance targets and have been implemented in health systems across low and middle-income countries (LMICs) [1].

To better align incentives offered with patient and population well-being (or health—one of the well-being arguments), “Pay for performance” schemes have become increasingly common in health care delivery in developing countries. In principle, the idea is simple: use the logic of pay for performance in human resource management [2, 3]. Use of performance incentives in wealthy countries began in earnest during the 1990s with programs that rewarded both process indicators and measures of clinical quality [4]. Examples of performance targets include immunization rates [5, 6] disease screening [7]; adherence to clinical guidelines [8]; and the adoption of case management processes, physician reminder systems, and disease registry systems. Many pay for performance schemes have attempted to directly link payment to quality of care [9].

The premise of P4P is that providers are responsive to financial incentives [10–14] and that each of the commonest payment methods (i.e., fee-for-service, capitation, and salary) is not designed to stimulate good performance and separately creates incentives for undesired behavior. Given that performance measurements have become more accurate over the past two decades, it therefore seems appropriate to use financial incentives explicitly to stimulate improvements in performance [15].

P4P programs are designed to align payments with organizational goals, which give financial incentives to service providers based on performance improvement based on specific criteria, such as quality improvement or cost control or providing services in deprived areas [16–18].

A variety of terms have been used interchangeably for pay-for-performance (P4P) incentives, including performance-based incentives (PBI), performance-based pay (PBP), and performance pay (PP). In the broadest sense of the term, P4P can be defined as a payment made to a national or subnational government body, to a health facility or any other healthcare provider, or even to a consumer of health services,3 once predefined results have been attained and verified [19]. This approach targets either the demand or the supply side of health service provision. If the latter is the case, it implies the transfer of financial incentives to health institutions and their staff according to an established ‘performance contract’ with a set of specific ‘performance indicators’ [20].

Pay-for-performance initiatives, if they are effective, can prevent the excessive growth of costs through the prevention and management of chronic diseases while maintaining efficiency, and also these initiatives have the potential to reshape the medical landscape by incentivizing physicians to concentrate on clear goals for common diseases [21].

P4P programs have many differences in definition and implementation. Most of the difference in P4P programs comes from the difference in the definition of indicators and dimensions of performance that are rewarded [22]. P4P has been implemented in the health care system of many countries. In the United States, P4P has been widely implemented in both the private and public sectors. In 2004, the UK introduced P4P in general medical contracts. Other countries such as: Australia, New Zealand, Costa Rica, Haiti, Nicaragua and Taiwan and Turkey have also used similar P4P programs for reimbursement in different programs [22].

All P4P programs include a common set of four basic elements, with a wide variety of choices made within those elements to meet different objectives. the common elements include:

I. performance domains and measures;
II. basis for reward or penalty;
III. nature of the reward or penalty;
IV. data reporting and verification [23, 24].

Therefore, there is always the question of which model is suitable to achieve better results for p4p implementation and what are the requirements to achieve the policy goals. The purpose of this study is to compare pay for performance models in different countries.

## Method

In this Descriptive-comparative study, pay for performance models in selected countries in 2022 have been compared. The method used for the comparative study was George Brady’s comparative research method. Brady’s model includes four stages: description, interpretation, comparison and comparison [25]. For the division of the research community, the model of the key frameworks of the health care system and their classification was used. Various models have been introduced for the classification of health care systems [26–33]. One of the most complete typologies divides the world’s health systems into 4 groups [34]. Table 1 shows this typology.

**Table 1:** Typologies of health systems

		Provision of health care	
		Public	Private
Financing administration	Single /concentrated	NHS (England-New Zealand)	NHI (Australia-Canada)
	Multiple/dispersed	SHI (Germany-France) SHI	Liberal model (US)

Based on the above classification, the countries of England, New Zealand, Germany, France, Australia, Canada and the United States: California were selected for comparison in this study. The reason for choosing these countries was to have successful experiences and policies in implementing the P4P pay for performance program in the primary health care sector and to have valid evidence available in the countries.

Required data in scientific databases PubMed, Web of Science, Embase/Medline via Ovid, Cochrane Library, Cochrane Central Registry of Controlled Trials, ProQuest and Scopus, Emerald, as well as the website of the World Health Organization (WHO), the World Bank and OECD library website, were searched using keywords related to payment methods, pay for performance (p4p) and financial incentive in primary care in selected countries. For this reason, in order to identify different P4P programs in selected countries and compare them, the standard framework of the P4P model, which including performance domains and measures, basis for reward or penalty, nature of the reward or penalty and data reporting, was used.

## Findings

In this research, the standard framework of the P4P model, which includes 4 elements, which are dimensions and performance indicators, payment strategies, the nature of incentives, and reports, has been used to compare different P4P programs in selected countries. A summary of these elements of P4P model is shown in Table 2. Then, the features of each country’s P4P program are briefly described.

**Table 2:** Summary of P4P model elements in selected countries

Country name and program name	Performance domains and measures	basis for reward or penalty	nature of the reward or penalty	data reporting and verification
**England** QOF	Clinical domain: Atrial fibrillation, secondary to coronary heart disease, heart failure, hypertension, peripheral arterial disease, stroke and TIA, diabetes, asthma, chronic obstructive pulmonary disease, Alzheimer’s disease, mental health, cancer, chronic kidney disease, epilepsy, disabilities, osteoporosis, rheumatism, palliative care, civilian hyperglycemia. • Public health domain Blood pressure, obesity, smoking, vaccination and belief, cervical screening • Quality improvement domain Prescription Drug Dependency, Optimizing Access to General Practice	Each indicator has a point value. The value of one QOF point for 2021-22 is £194. 83..	*Absolute* Per cent of target met after minimum threshold is reached	Electronic health record
**New Zealand** PHO	Chronic disease screening • Breast cancer coverage, cervical cancer screening coverage, ischemic heart disease diagnosis, heart disease risk assessment, diagnosis, post-diagnosis diagnosis, smoking status, Western advice or support to quit smoking	Per cent attainment of target	*Absolute*	Electronic health record
	Prevention of infectious diseases • Influenza vaccination in the elderly (over 65 years), percentage of children fully vaccinated.			
**Germany** DMP	• • Documentation and coordination • Information, consultation, registration and preparation of initial documents, preparation of draft follow-up documents • • Follow up of patients • Continuity of care and treatment of patients with type 2 diabetes • • Additional services • Comprehensive consultation for diagnosis of diabetic neuropathy, care of diabetic foot lesions in each foot, referral to nephrologist, eye exam documentation • • Training fee • Treatment and educational program for patients without insulin therapy (four sessions with a maximum of four patients in four weeks), auxiliary materials for education (without diabetes license)., treatment and educational program for patients with high blood pressure.	Flat rate for participation and per service	*Absolute*	Claims data
**France** ROSP	• Prevention and screening Influenza immunization (2 indicators), breast cancer screening (1 indicator), cervical cancer screening (1 indicator), prescription of vasodilator drugs for elderly patients (1 indicator), prescription of long half-life benzodiazepines (2 indicators), antibiotics therapy ( 1 index) • Chronic disease management diabetes (8 indicators), blood pressure (1 indicator), •Cost- effective prescribing Antibiotics (1 index), PPIs (1 index), statins (1 index), antihypertensive drugs (1 index), antidepressants (1 index), ACEI/ARBs (1 index), aspirin (1 index)	Achievement rate- progress toward target relative to baseline performance	*Absolute*	Claims data
	• Practice organization Updating the electronic file system (1 indicator), approved prescription software (1 indicator), computer equipment and software for online consultation (1 indicator), notification through the clinic website (1 indicator), annual evaluation of medical records electronic patient, and providing a combined report to the patient (1 indicator)			
**Australia** PIP	Quality stream Quality Prescribing, Diabetes Incentive, Cervical Screening Incentive, Asthma Incentive, Indigenous Health Incentive Capacity stream eHealth Incentive, Practice Nurse Incentive, After Hours Incentive, Teaching Incentive, Aged Care Access Incentive Rural support stream Rural Loading, Procedural GP Payment, Domestic Violence Incentive	Flat rate for participation, targets, and per patient reached	*Absolute*	Claims data
**Canada** FHO	Cumulative preventive care Influenza vaccination for people over 65 years old, children vaccination, cervical cancer screening (Pap smear), breast cancer screening (mammography), colorectal cancer screening) • Additional service incentives After-hours care, newborn care, congestive heart failure, smoking cessation counseling, maternity services, palliative care, home visits (other than palliative care), long-term care, Laboure and Delivery, Office Procedures, prenatal care, hospital services Special, primary health care for patients with serious mental illnesses	Per cent attainment of target	*Absolute*	Claims data
**US – California** Integrated Healthcare Association (IHA) Program	Clinical Quality 1.Cardiovascular 2.Diabetes Care 3.Musculoskeletal 4.Prevention 5.Respiratory	*Varies by insurer*	*Varies by insurer*	Claims data
	Meaningful Use of HIT			
	1. Use CPOE for medication orders.			
	2. Implement drug- drug and drug- allergy interaction checks			
	3. Maintain up- to- date problem list of current and active diagnoses			
	4. Generate and transmit permissible prescriptions electronically (eRx)			
	5. Maintain active medication list			
	6. Maintain active medication allergy list			
	7. Record demographics			
	8. Record and chart changes in vital signs			
	9. Record smoking status			
	10. Report ambulatory clinical quality measures			
	11. Implement one clinical decision support rule relevant to specialty or high clinical priority, along with the ability to track compliance with that rule			
	12. Provide patients with an electronic copy of their health information			
	13. Provide clinical summaries for patients at each office visit			
	14. Capability to exchange key clinical information			
	15. Protect electronic health information created or maintained by the certified EHR technology			
	16–20. Any (5) CMS/ONC Menu set measures			
	21. Chronic Care Management for Diabetes, Depression and one other Clinically Important Condition			
	22. Within- PO Performance Variation			
	Patient Experience			
	1. Doctor–Patient Interaction Composite for PCPs			
	2. Doctor–Patient Interaction Composite for Specialists			
	3. Coordination of Care Composite			
	4. Timely Care and Service Composite for PCPs			
	5. Timely Care and Service Composite for Specialists			
	6. Overall Ratings of Care Composite			
	7. Office Staff Composite			
	8. Health Promotion Composite			
	Appropriate Resource Use			
	1. Inpatient Utilization: Acute Care Discharges PTMY			
	2. Inpatient Utilization: Bed Days PTMY			
	3. Inpatient Readmission Within 30 days			
	4. Emergency Department Visits PTMY			
	5. Outpatient Procedures Utilization: per cent Done in Preferred Facility			
	6. Generic Prescribing: SSRIs/SNRIs			
	7. General Prescribing: Statins			
	8. Generic Prescribing: Anti- Ulcer agents			
	9. General Prescribing: Cardiac- Hypertension and Cardiovascular			
	10. Generic Prescribing: Nasal Steroids 11. General Prescribing: Diabetes – Oral 12. Generic Prescribing: Anxiety/Sedation – Sleep Aids 13. Total Cost of Care 14. Frequency of Selected Procedures – Back Surgery 15. Frequency of Selected Procedures – Total Hip Replacement 16. Frequency of Selected Procedures – Total Knee Replacement 17. Frequency of Selected Procedures – Bariatric Weight Loss Surgery 18. Frequency of Selected Procedures – PCI 19. Frequency of Selected Procedures – Carotid Catheterization 20. Frequency of Selected Procedures – CABG 21. Frequency of Selected Procedures – Cardiac Endarterectomy			

### England: quality and outcomes framework (QOF)

The NHS model is widely regarded as the international best practice in delivering health services focused on primary care, and the focus on primary care has helped to contain the cost and efficiency of the system. Almost all GPs in the UK are private entities contracted by primary care organizations under the NHS [23]. Payment to the general practitioner or the family physician is a combination of capitation and pay for performance [35].

The P4P program of the National Health System, known as QOF. QOF established process standards of surveillance for patients with chronic diseases. Performance was measured in terms of successful periodic review and control of conditions such as high blood pressure and diabetes. Performance was rewarded with points for successively high levels of coverage, and each point had a monetary value. The QOF bonuses were paid to each GP practice, thereby incentivizing collective behavior. The effects of the QOF were to improve mean performance and reduce dispersion [36]. In this program, contracts were considered for a 25% increase in the salaries of general practitioners depending on their performance based on quality indicators. The participation of the contracting parties in the QOF program is voluntary [37, 38].

QOF incentives include three main areas known as domains. which include: Clinical domain; Public health domain and quality improvement domain. Each domain includes a set of progress measures, known as indicators, and activities are scored according to their level of success. QOF 2020-21 measures achievement with 68 indicators and a total of up to 567 points can be obtained based on achievement against each indicator.

Clinical domains: includes 57 indicators in 20 clinical fields (such as chronic kidney disease, heart failure, and high blood pressure) with a maximum point of 401.

Public health domains: includes eleven indicators (with a maximum value of 160 points) in five areas of blood pressure, obesity and smoking, vaccination and immunization, and cervical cancer screening.

Quality improvement: includes four indicators (with a maximum value of 74 points) in two areas - Prescription Drug Dependency and Optimizing Access to General Practice [39].

The final payment is adjusted taking into account the workload, local demographics, and the prevalence of chronic diseases in the practice area [35]. Each index has a maximum point value. The value of one QOF point for 2021-22 is £194/83. The points are distributed in such a way that more weight is assigned to the indicators with more workload and more connection with the results [39]. In England, payments are made directly to practices, which are generally groups of 1 to 10 primary care physicians. These payments include 25 to 30% of primary care income [40].

### New Zealand: primary health organization performance program (PHO)

New Zealand has a predominantly (about 80%) publicly funded healthcare system, which is mainly funded by general taxation. Funding is devolved to 20 District Health Boards (DHBs) which manage, purchase and/or provide health and disability services for their geographically defined populations. DHBs fund primary care through Primary Health Organizations (PHOs) who contract with GPs and other non-government providers to provide services [41]. General practitioners can join PHO voluntarily. Patient registration in PHO is also not mandatory, but GPs and primary health organizations must have an official registered patient list to be eligible for government subsidies. Patients must register with a general practitioner of their choice. General practitioners act as the gatekeepers of specialized care [42].

The PHO program includes a set of eleven performance indicators in the two areas of chronic patients and vaccination. The program also compiles a set of indicators that are for information only and are not linked to incentive payments. Some indicators are measured separately for “high-need populations” and are rewarded at a higher rate. The high-needs population is defined as the sum of individual registered patients who are Māori (New Zealand’s indigenous population), Pacific Islanders or live in geographic areas of relative socioeconomic deprivation. To strengthen incentives to reduce health disparities, payments for performance are heavily weighted when measuring progress and outcomes among high-needs populations [23, 43].

Fixed payments for most indicators are made to the PHO for each six-month performance period based on the percentage of achievement of each target. Performance pay amounts are based on the following:

- Population enrolled in PHO for performance period.
- Progress toward goals for each performance indicator.
- The amount of payment defined in the PHO contract for each performance period for each registered person.

But total incentive payments account for less than 1% of government primary care spending [23].

### Germany: disease management programs (DMP)

The German health system is the Bismarck model. The Ministry of Health pays the resources collected through general tax, insurance tax and private and government insurance premiums to statutory health insurance (SHI) and supervises it. In Germany, the various levels of government play almost no role in directly financing or providing health care. This responsibility is mainly delegated to independent associations in insurance funds and provider associations, which are jointly represented by the Federal Joint Committee [44]. The pay for performance program in Germany is called “Disease Management Programs (DMPs)”, which was introduced in Introduced in 2002, DMPs have since been implemented to position primary care physicians as care coordinators for patients with chronic diseases, using financial incentives to reward better quality of care. Health funds can design their own DMPs. There are differences in the organizational features of DMPs in different regions, as health funds separately define the organizational arrangements and implementation of DMPs. Health funds receive incentives to create DMPs and enroll patients, and in turn provide incentives to physicians [23]. Reimbursement for family physicians who are members of this program is done through a combination of merit payment methods and additional bonuses based on the provision of specific services (such as prevention) [45]. DMPs now cover six major chronic disease areas: diabetes - type I and II, asthma, chronic obstructive pulmonary disease, coronary heart disease, and breast cancer [23].

### France: payment for public health objectives (ROSP)

France has compulsory health insurance. Private medical insurance serves to cover additional services and also as excess cost coverage [46]. In France, an experimental measure based on voluntary participation, the Contract for Improvement of Individual Practice (Contrat d’Amélioration des Pratiques Individuelles—CAPI), was launched in 2008 to introduce payment by capitation into the remuneration of general practitioners (GP). In 2011, this measure was extended and became Remuneration based on Public Health Objectives (Rémunération sur Objectifs de Santé Publique—ROSP). ROSP applies to GPs as well as to certain specialists and is regularly updated. Currently, it includes 29 clinical indicators for GPs caring for adult patients. This P4P approach rewards all GPs by providing additional payments based on the level of achievement of ROSP indicators, as assessed by quality indicators. The list of indicators is known, so GPs can consult the expected performance criteria for this additional source of income [47].

### Australia: practice incentives program

The insurance system of Australia is National Health Insurance (NHI). The method of payment in the primary care sector in this country is often in the form of FFS (95% of the payment of family physicians) and 5% pay for performance, and there is no capitation payment in this sector [48]. The pay for performance program in This country is called " Practice Incentive Program - PIP” which has been implemented in this country since 1998. The main goal of PIP is to encourage continuous improvement in primary care through financial incentives to support quality care and improve access and health outcomes for patients. And a total of 13 indicators rewards the performance of general practitioners [6].

### Canada: family health organizations (FHOs)

Canada has government health insurance specific to each state and the central government supports national programs through Medicare health insurance. The dominant model of primary care across Canada has traditionally been based on solo or group practice physicians, with reimbursement primarily through the submission of FFS bills to provincial health plans for eligible services. Since 1999, the province of Ontario has introduced pay-for-performance incentives for a range of preventive primary care services and some other services provided by family physicians with the aim of improving the quality of patient care. These primary care reforms have been introduced in the form of 4 different models (FHN(2002), FHG(2003), CCM(2005), FHO(2006)) [48, 49].

Family Health Organizations (FHOs), which started in 2006, consist of at least 3 doctors and their financing is based on a combined per capita. Older models of per capita payments were based on age and sex, but in this model, per capita is based on Health care needs or social inequalities are regulated. This model includes 5 main functional areas (influenza vaccination for people over 65 years old, children vaccination, cervical cancer screening (doing pap smear), breast cancer screening (doing mammography), colorectal cancer screening) and 3 activity areas after working hours. Smoking cessation counseling and special care. How to pay incentives If the target threshold is reached, a fixed amount of bonus is paid [49].

### United States: California integrated healthcare association physician incentive program

The federal government is responsible for organizing and regulating the health care system in the United States. The financing of the United States is carried out through the collection of general taxes and advance payments. These revenues are allocated to Medicare, Medicaid, and health plans to pay general practitioners (GPs), family physicians, and specialists [50]. The payment mechanism by Medicare and Medicaid, as well as managed care payments, is fee-for-service. Health maintenance organizations (HMOs) and preferred provider organizations (PPOs) pay physicians fee-for-service (FFS) and per capita. Some large HMOs pay physicians a salary [51]. In general, in the United States, insurers and health plans use fee-for-service (FFS), capitation, and fees to pay primary care physicians and specialists. they do. “Pay-for-performance (P4P)” also rarely makes up more than 5% of an America physician’s pay. Out-of-pocket payments (OOP), insurance co-payments, co-payments, and VAT have also increased significantly in recent years for health services [50]. One of the first and perhaps the largest private pay-for-performance (P4P) initiatives was launched by the Integrated Healthcare Association (IHA) in 2001 with Eight health plans representing ten million members were launched in California, USA.

In California, many primary care physicians participate in larger medical organizations (such as multispecialty medical groups and independent practice associations, or IPAs) that contract with health plans on their behalf, and incentive payments to these larger organizations instead of individual physicians [40]. In the California program, there are four domains or functional areas that are recommended for use in P4P, including clinical quality, health information technology, patient experience, and resource utilization, and in It includes a total of 78 indicators. Consolidated performance results are used by health plans to calculate bonuses distributed each year. Each P4P funding plan determines its own methodology for calculating bonus payments to physician groups. IHA proposes a standardized payment method, in which physician groups are scored on the extent of achievement and improvement for each measure [23]. Table 3 shows Payment characteristics of primary care physicians in selected countries and Table 4 shows the Summary of objectives for P4P programs in primary care.

**Table 3:** Payment characteristics of primary care physicians in selected countries

country name	Individual goal setting/group goal setting	Method of payment to general practitioners
**England**	group	capitation ، FFS, P4P
**New Zealand**	group	capitation ، Patient participation ، P4P
**Germany**	Individual	capitation ، P4P
**France**	Individual	FFS, P4P
**Australia**	Individual and group	FFS, P4P
**Canada**	Individual	capitation ، FFS, P4P
**United States: California**	Individual and group	capitation ، salary ، FFS, P4P

**Table 4:** Summary of objectives for P4P programs in primary care

country name	Prevention of chronic diseases	Cancer screening	vaccination	Efficiency and optimal use of resources	Smoking cessation advice	Use of information technology and electronic health record	mental health	Providing service After-hours care	Supporting rural and remote areas
**England**	✱	✱	✱	✱	✱		✱	✱	
**New Zealand**	✱	✱	✱		✱				✱
**Germany**	✱	✱				✱			
**France**	✱	✱	✱	✱		✱	✱		
**Australia**	✱	✱		✱		✱		✱	✱
**Canada**	✱	✱	✱		✱		✱	✱	
**United States: California**	✱	✱	✱	✱	✱	✱	✱		

## Discussion

Our goal in this study was to compare p4p programs in selected countries using the four main components of the p4p model [22].

The results of this study showed that Most of the difference in P4P programs comes from the difference in the definition of indicators and dimensions of performance to which the reward is awarded.

in general, indicators can be divided into clinical and non-clinical categories. In the category of clinical indicators, which includes prevention of chronic diseases, cancer screening, vaccination, the functional area was common in all P4P programs of the studied countries, although Australia and Germany lacked payment incentives in the field of vaccination and safety. Some countries have set clear goals for the efficiency and optimal use of resources in their p4p program and by using indicators such as Prescription Drug Dependency (England), Quality Prescribing (Australia), Cost- effective prescribing (France), Appropriate Resource Use (United States: California) has addressed this issue.

In the case of non-clinical indicators, performance measures generally reflect the use of information and communication technology (ICT) (i.e., for registration, appointments and other facilities and the use of electronic health records), which in the German indicators There are France, Australia and California.

Increasing access to services is one of the important goals for countries, which is included in the indicators of some countries such as New Zealand, which have specified some indicators aimed at reducing health inequalities separately for “high-needs populations”. Some programs in New Zealand and Australia have tried to reduce inequality in access to services by providing incentives to “deliver services to remote and rural areas”.

Optimizing access to local primary health care is a complex and long-term challenge that has been exacerbated under the pressures of the coronavirus pandemic. A review of access to GPs during the pandemic by Healthwatch England showed that some people are having difficulty booking appointments and accessing treatment [52]. The same England in the latest edition of the QOF-2022 program Optimizing Access to Public Care [52] In addition, Australia and Canada also have incentives in their programs to provide after-hours services, which improves people’s access to primary care and family doctor services.

Overall, the literature highlights that P4P schemes quite often have various positive effects/impacts on intermediate results (inputs, outputs, and less often, outcomes), although many mixed results are reported in the literature. But it is important to bear in mind that not all studies have the same methodological robustness (measurement of effects and consideration of confounding factors) [19]. P4P schemes improve access to and use of health facilities, antenatal and childbirth care dispensed by qualified staff in health facilities, the promotion of family planning activities, the diagnosis and treatment of malaria, HIV, and tuberculosis, and also the implementation of protocols to improve the quality of care [53–67].

diversity of this program, it makes it difficult to compare and evaluate, but in general, the selection of indicators In each country, depending on the health priorities of each country, the health and treatment system of that country tries to increase the quality and quantity of those services by providing rewards and incentives, for this reason, indicators related to mental health (England, France, Canada and California) and smoking cessation counseling (England, New Zealand, Canada and California.) are included as indicators in the P4P program. Overall, The studies conducted to design a successful P4P recommend the use of process (intermediate) and result indicators as target criteria [68].

The second element of a P4P program is the basis for reward or penalty, or how achievement against performance indicators is used to determine the level of the incentive payment earned by the provider. The most common options include: the absolute level of the measure (e.g., whether a target was achieved or the number of patients reached); the change in the performance measure (improvement), or how the provider performs against the measure relative to other providers (relative ranking) [69]. In all P4P programs (Except for California), service providers use absolute metrics. In such a way that for each index one target threshold (such as New Zealand and Canada) or a certain point or a fixed amount for the realization of that index (England, Germany, France, Australia) they have considered.

The third common element of P4P programs is the reward or penalty, which may be financial or non- financial, or a combination of both. In all the studied countries, financial incentives were used for encouragement. The effects of financial (bonuses and penalties) and nonfinancial incentives (reputation and peer pressure) are difficult to separate. An important problem with the UK QOF is whether the behavior change was a product of the bonuses paid or the comparative performance measurement that affects the reputation of clinicians and institutions. Those designing P4P incentive systems should ensure that not only can the relative effects of financial and nonfinancial interventions be identified but also that their reforms enhance and do not erode nonpecuniary incentives such as duty, trust, and reputation [36].

Also, the effect of incentives on provider behavior may be influenced by whether payments are made to individual providers or at the group/organizational level. Given the increasingly collaborative forms of care delivery, a collective effort is needed to improve primary care practice. Therefore, holding individual providers accountable for performance is less valid, especially for team-based care. However, at the same time, if incentives are paid at the group level, it is essential that they also reach individual care providers, otherwise they may not influence behavior [70]. There is some evidence that the best focus of incentives is the clinical team. For instance, the NHS incentive scheme for general practitioners in primary care was conditional on practice or team performance and improved activity performance [36]. A counter-argument is that P4P incentive schemes should be focused on the institution rather than the individual physician as that is where the financial risk lies. This is true if institutional budgets are not devolved to clinical teams. If there is budget devolution, teams may respond positively to clinical and financial pressures to improve the efficiency of patient care [36, 71].

In the countries under study, only England, New Zealand have group payments, the United States (California) and Australia also have both individual and group payments.

The next important issue that has been investigated in this study is how to pay general practitioners or family physician in P4P programs. The results of this study show that all selected countries have implemented a combination of payment systems. The “mixed payment system” may reduce the quantity and improve the quality of health care compared to the FFS model and can provide the necessary incentives to achieve the goals of preventive care [72]. An important part of the payment of general practitioners in most of the studied countries, including Canada, New Zealand, England, Germany and United States: California, is the per capita method. These countries adjust per capita by various variables such as age, gender, pregnancy and socio-economic status in the enrolled population. The highest adjustment coefficient per capita has been done in New Zealand, England. The variables used in the per capita adjustment should depend on the health indicators and the socio-economic level of the enrolled population. For example, the capitation payment in England takes into account not only the patient’s age and sex, but also their health status, while New Zealand also takes into account the patient’s use of health services. Capitation based on the patient’s health status and need for care incentivizes physicians to treat harder-to-care patients without requiring additional premiums.

Access to data related to indicators is the fourth component of the P4P program and a main determinant in the design of the P4P program and in improving performance. The report of indicators may be mainly based on claimed data (self-reporting) or electronic registration of the patient’s file. Claims data includes billing codes that physicians, pharmacies, hospitals, and other health care providers send to payers (e.g., insurance companies, Medicare, etc.) [73]. In the studies, only England and New Zealand use data recorded in electronic patient records, and other countries use claims data to report indicators.

Small practices, where the majority of patients still receive care nationally, historically have provided lower quality care—especially solo practices—and may have greater obstacles to improving care because they have lacked the scale and organizational structure to do so. With widespread implementation of EHRs, it is possible that EHR-enabled solo and small group practices will be able to respond to P4P incentives and improve quality [74].

## Conclusion

P4P is now widely used in many primary health care systems around the world, but overall, the evidence for the effectiveness of P4P for improving quality of care in primary care is mixed. This is to some extent due to the fact that the P4P schemes used in primary care vary considerably. There are many different schemes that incentivize different aspects of care, in different ways and in different settings. This makes evaluation problematic. By providing a general framework, this study investigated the characteristics of P4P programs along with the conditions and context of different countries. Because the same incentives can lead to very different effects, because their impact depends on a variety of factors, from the socio-economic and cultural context of the health care system and the health care goals of governments to the personal characteristics of the health care provider.

The general framework developed in this study can be useful for target users who are developing and evaluating a P4P application. Careful attention to P4P design is important because poorly designed programs may lead to undesirable provider behavior.

## Data Availability

The datasets used and/or analyzed during the current study are available from the corresponding author upon reasonable request.
